# Deep learning-based single image super-resolution for low-field MR brain images

**DOI:** 10.1038/s41598-022-10298-6

**Published:** 2022-04-16

**Authors:** M. L. de Leeuw den Bouter, G. Ippolito, T. P. A. O’Reilly, R. F. Remis, M. B. van Gijzen, A. G. Webb

**Affiliations:** 1 Delft Institute of Applied Mathematics, Delft University of Technology, Delft, The Netherlands; 2 Department of Metrology, ASML Netherlands, Veldhoven, The Netherlands; 3 C.J. Gorter Center for High-Field MRI, Leiden University Medical Center, Leiden, The Netherlands; 4 Circuits and Systems, Delft University of Technology, Delft, The Netherlands

**Keywords:** Computational science, Magnetic resonance imaging

## Abstract

Low-field MRI scanners are significantly less expensive than their high-field counterparts, which gives them the potential to make MRI technology more accessible all around the world. In general, images acquired using low-field MRI scanners tend to be of a relatively low resolution, as signal-to-noise ratios are lower. The aim of this work is to improve the resolution of these images. To this end, we present a deep learning-based approach to transform low-resolution low-field MR images into high-resolution ones. A convolutional neural network was trained to carry out single image super-resolution reconstruction using pairs of noisy low-resolution images and their noise-free high-resolution counterparts, which were obtained from the publicly available NYU fastMRI database. This network was subsequently applied to noisy images acquired using a low-field MRI scanner. The trained convolutional network yielded sharp super-resolution images in which most of the high-frequency components were recovered. In conclusion, we showed that a deep learning-based approach has great potential when it comes to increasing the resolution of low-field MR images.

## Introduction

High-field MRI scanners, which are the norm in clinical settings, utilize fields in the order of several tesla. To generate these strong and homogeneous fields, superconducting magnets are required, which accounts for the cost, size and infrastructure demands of high-field MRI scanners. Therefore, access to MRI technology is limited for people residing in low- and middle-income countries^1^.

To increase the accessibility of MRI scanners, the development of low-field MRI scanners is of great clinical relevance. Examples of such scanners are described by Cooley et al.^2^, Obungoloch et al.^3^, O’Reilly et al.^4^, Ren et al.^5^ and Tsai et al.^6^, among others. Low-field scanners do not depend on superconducting magnets and are therefore significantly more reasonably priced. Additionally, due to their less stringent infrastructure demands, they can be made portable, which would enable their transport to populations living in rural areas in low- and middle-income countries. For a review on low-cost and portable MRI, the reader is referred to Wald et al.^7^ An overview on MR physics in low-field MRI was provided by Marques et al.^8^.

The low-field scanner described by O’Reilly et al.^9^ was developed as part of a project that aims to construct a platform to image pediatric hydrocephalus in developing countries. Hence, the scanner was designed to accommodate infants’ heads. In general, scan times are long in MRI and a significant amount of contemporary MRI research focuses on accelerating the acquisition process. Clearly, staying still for dozens of minutes on end is even more challenging for infants than for adults, and hence efforts should be taken to make the scan duration as short as possible, while maintaining good image quality.

Assuming a full sweep of the spatial frequency domain, i.e., k-space, a shorter scan duration corresponds to an image of a lower resolution. Super-resolution image reconstruction techniques attempt to reconstruct a high-resolution (HR) image from one low-resolution (LR) image or several LR images^10^. We will focus on single image super-resolution (SISR), in which case we only have one LR image at our disposal. SISR is an ill-posed problem, because one LR image can correspond to several different HR images, and hence it does not have a unique solution.

We note that, to decrease the scan time and still reconstruct an image of good quality, one could also resort to compressed sensing (CS) techniques^11^ by sampling only part of k-space and assuming that the image is sparse in some transform domain. However, we will focus on super-resolution (SR) image reconstruction instead. This decision was based on the observation that the SR problem is more straightforward than the CS problem: in SR, we can work with the low-resolution images, while in CS, we work with the k-space data instead. Additionally, the k-space sampling pattern has to be taken into account in CS. We will use a deep learning technique to improve image quality and, after being trained on LR-HR image pairs, our neural network should be applicable to all low-field MR brain images of the resolution the network was trained on.

One class of methods that can be used for SISR relies on interpolation, like bicubic interpolation^12^, Lanczos interpolation^13^ and zero-padding the k-space data, the latter of which is common for MRI images. These methods are efficient and straightforward but the results they provide are generally lacking in accuracy when it comes to recovering high-frequency components^14^. Reconstruction-based SR methods generally pose the problem as a minimization problem in which prior knowledge about the solution is incorporated, thereby restricting the solution space^15–19^. Drawbacks of reconstruction-based methods are that they are generally computationally expensive and that they require the tuning of hyperparameters. Learning-based (or example-based) methods form the third class of methods that can be used to tackle the problem of SISR. In general, these methods use machine learning to extract relationships between LR images and their HR counterparts. They are computationally fast and usually perform well^14^. Examples include methods based on Markov random fields^20^, sparse representations^21,22^ and neighbor embeddings^23^. Of the learning-based methods, methods using deep learning (DL) generally outperform all other reconstruction-based and learning-based methods^14^. Examples of convolutional neural networks that have been used for SISR are SRCNN^24^, SRDenseNet^25^ and SRGAN^26^, among many others. For a more complete overview of the DL methods that have been used for super-resolution, the reader is referred to Wang et al.^27^.

Deep learning has been applied to the problem of SISR in MRI before. Pham et al.^28^ applied a 3D version of the SRCNN network to MR brain images. Similarly, Chen et al.^29^ developed DCSRN, a densely connected convolutional network, which is closely related to a 3D version of SRDenseNet, that was trained to carry out super-resolution on 3D MR brain images as well. Masutani et al.^30^ applied modified versions of the SRCNN architecture and the U-Net^31^ architecture to cardiac MRI images. Chen et al.^32^ trained a multi-level densely connected network using a generative adversarial network (GAN)-based loss function.

In this work, we employ a convolutional neural network of the SRDenseNet architecture^25^ to carry out SISR on MR brain images. The main contribution and the novelty of our work is in the application: we focus on low-field MR brain images. Low-field MRI is a rapidly growing field with a lot of potential, but it also comes with challenges. This paper demonstrates the potential of deep learning-based methods in addressing the challenges in low-field MR imaging.

## Methods

### Background

Given a low-resolution 2D image ${\mathbf {y}}$, our aim is to acquire its high-resolution counterpart ${\mathbf {x}}$. The relationship between ${\mathbf {x}}$ and ${\mathbf {y}}$ can be modeled as follows:

1 $$\begin{aligned} {\mathbf {y}}= {\varvec{{\mathscr {F}}}}_{LR}^{-1}{\mathbf {D}}{\varvec{{\mathscr {F}}}}_{HR}{\mathbf {x}}+ {\mathbf {n}}, \end{aligned}$$

where ${\varvec{{\mathscr {F}}}}_{LR}^{-1}$ is the inverse FFT operator applied in the LR regime, ${\mathbf {D}}$ is an operator that selects only the low-frequency components in k-space, which, in our case, yields a matrix of size $64\times 64$, ${\varvec{{\mathscr {F}}}}_{HR}$ is the FFT operator for the HR regime ($128\times 128$) and ${\mathbf {n}}$ is an (unknown) noise vector. The goal of super-resolution is to find an approximate inverse of the operator ${\varvec{{\mathscr {F}}}}_{LR}^{-1}{\mathbf {D}}{\varvec{{\mathscr {F}}}}_{HR}$. We note that the standard super-resolution problem is generally posed differently, i.e., the HR image is assumed to undergo blurring and downsampling, culminating in an LR image. However, using Eq. (1) follows the low-resolution MRI acquisition process more accurately.

### Convolutional neural network

**Figure 1:**
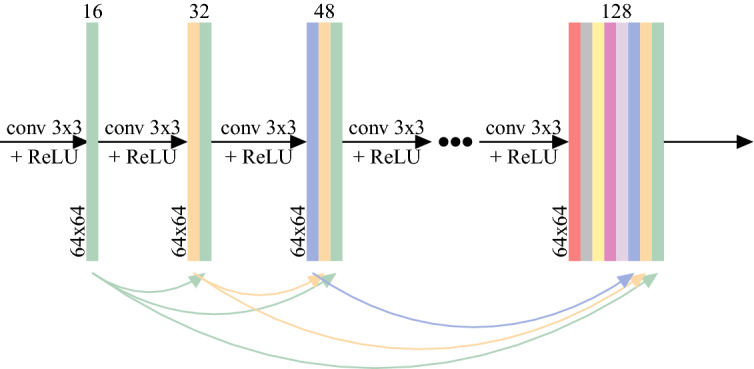
A dense block, which is a fundamental component of the SRDenseNet architecture, contains eight convolutional layers that receive the outputs of all preceding layers as input.

**Figure 2:**
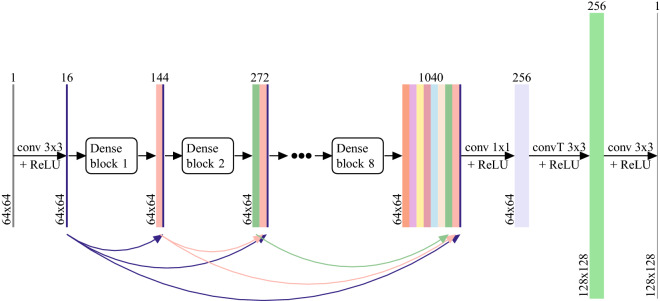
The SRDenseNet convolutional neural network^25^ that is used to carry out single image super-resolution on low-field MR images.

We chose a convolutional neural network of the SRDenseNet architecture for our application. Our choice was motivated by SRDenseNet’s good performance, combined with its manageable number of parameters. We note that, as there is a vast literature on deep learning-based methods for super-resolution, other networks may be applicable as well^24,26,27^. SRDenseNet was introduced by Tong et al.^25^ It consists of blocks of densely connected convolutional layers (“dense blocks”). In every dense block, which is consistent with the DenseNet architecture^33^, each convolutional layer receives as input the concatenated outputs of all preceding convolutional layers, as shown in Fig. 1. By reusing feature maps in this way the learning of redundant features is avoided. Instead, the current layer is forced to learn supplemental information. As in the original paper, we will use 8 dense blocks of 8 convolutional layers each, where each convolutional layer produces 16 feature maps, which means that each dense block yields 128 feature maps. In each convolutional layer, the kernel size is 3x3. After the final dense block, a bottleneck layer with convolutional kernels of size 1x1 is used to reduce the number of feature maps to 256, followed by a transpose convolutional layer (which is often called a deconvolution layer) which upsamples the image to HR space. Note that in this work, the upsampling factor is equal to 2 and hence we use only one single transpose convolutional layer with a stride of 2, as opposed to the 2 transpose convolutional layers in the original SRDenseNet which was used for an upsampling factor of 4. Finally, another convolutional layer with a 3x3 kernel is applied to reduce the output to a single channel. All layers except for the final convolutional layer use a nonlinear ReLU (Rectified Linear Unit) activation function. Additionally, skip connections are employed to feed the output of each dense block to each of the subsequent dense blocks, as is consistent with the SRDenseNet_All architecture showcased in the original paper^25^. The complete architecture, which has 1,910,689 trainable parameters, is shown in Fig. 2.

### Dataset and training

In this work, we focused on 2D images, but it should be noted that this approach can be extended to 3D. We generated a training and validation set using 2D images obtained from the publicly available NYU fastMRI Initiative database (fastmri.med.nyu.edu)^34,35^. As such, NYU fastMRI investigators provided data but did not participate in analysis or writing of this manuscript. A listing of NYU fastMRI investigators, subject to updates, can be found at the aforementioned website. The primary goal of fastMRI is to test whether machine learning can aid in the reconstruction of medical images. The database consists of slices of T1-weighted, T2-weighted and FLAIR (fluid-attenuated inversion recovery) images, acquired using 1.5 T and 3 T MRI scanners. By training on such a variety of MR brain images, the resulting network should be applicable to images acquired using different sequences as well, without the need to re-train the network whenever the parameter settings change. We note that, even if we were planning on applying the trained network to, for example, T1-weighted low-field MR images only, it would still make sense to train the network on high-field MR images acquired using different kinds of sequences, making it adaptable to different kinds of input. The reason for this is that the relaxation times vary with field strength and hence, a T1-weighted image acquired using a low-field scanner might look different from one acquired using a high-field scanner. One parameter to be careful with, though, is the image size. We will use input images and output images of size $64\times 64$ and $128\times 128$, respectively. Because of the purely convolutional nature of the network, it is possible to use images of a different size as input. The network should be able to accommodate small deviations in size. However, it is unlikely that it would generalize to images that deviate significantly in size from the images in the training set.

**Figure 3:**
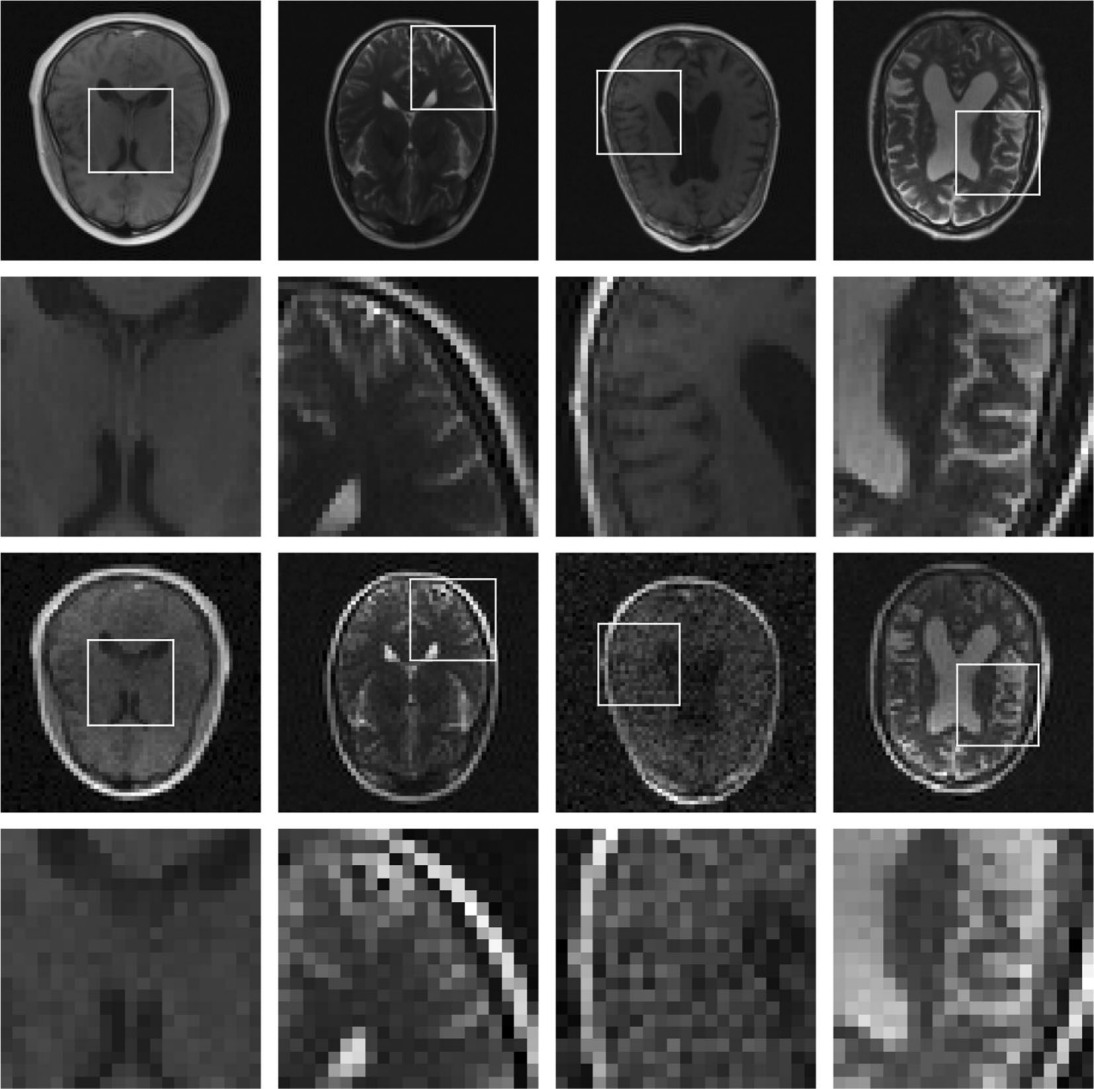
Examples of HR-LR image pairs in the training set. The first row contains four different HR images, with the white squares denoting patches whose zoomed-in versions are shown in the second row. In the third row, the corresponding (noisy) LR images are shown, with the fourth row containing LR versions of the patches in the second row.

The images in the database have different sizes. As we are interested in HR images of $128\times 128$ pixels, all images were resized to $128\times 128$ pixels. This was done by using an FFT to convert the images to k-space data, selecting the central part of k-space and subsequently applying an inverse Fast Fourier Transform (FFT), as in Eq. (1). After that, we downsample these HR images to LR images of $64\times 64$ pixels, by, again, using Eq. (1), i.e., we use an FFT to convert the image to k-space, select the central part of k-space (of size $64\times 64$) and apply an inverse FFT to obtain an LR image. To obtain noisy LR images, we add complex Gaussian noise in k-space, with the noise level varying from image to image. We used a range of noise levels consistent with the low-field MR images we have seen in practice. This step is necessary to make the convolutional neural network generalize to images acquired using a low-field MRI scanner, which, due to the weaker magnetic field, yields signals with a relatively low SNR^36^. In this way, 29,059 and 17,292 image pairs were obtained from the training and validation sets that are provided in the dataset, respectively. We assigned 10,000 of the 17,292 image pairs in the validation set to our own validation set, and the other 7292 to our test set. Some examples of image pairs present in the training set are shown in Fig. 3. We note that the data was split at the patient level, and hence, no data leakage occurred.

Since SRDenseNet is a purely convolutional neural network, it is possible to train on patches instead of complete images, which requires less memory during training. Furthermore, using patches allows us to generate more data. Therefore, we used the HR-LR image pairs to create 190,000 pairs of patches to train the network on, and 10,000 pairs of patches for validation, the HR and their corresponding LR patches having a size of $32\times 32$ pixels and $16\times 16$ pixels, respectively.

The convolutional neural network was implemented in TensorFlow^37^. The Adam optimizer^38^ with a learning rate of $10^{-3}$ was used to minimize the mean-squared error loss between the network output and the model HR image patches. Additionally, we investigated two different loss functions: $\ell _1$-loss and HFEN (High-Frequency Error Norm) loss^39^. However, after visual inspection of the resulting images, we found that the mean-squared error loss outperformed the others. We used a batch size of 20 and a total number of epochs of 74 because this corresponded to the smallest value of the validation loss. The training was carried out on a Titan X Geforce GPU (12GB) and took about 5 hours.

### Low-field MR image acquisition

Two three-dimensional in vivo scans of the brains of two healthy volunteers were acquired using the low-field MRI scanner described by O’Reilly et al.^9^ We will use different (2D) slices of the resulting 3D images as our network input. Both experiments were carried out using a turbo spin echo sequence. For the first experiment, the following parameters were used: FoV (field of view) $224\times 224\times 175$ $\hbox {mm}^3$, voxel size $1.75 \times 1.75 \times 3.5$ $\hbox {mm}^3$, $T_R$/$T_E$ (repetition time/echo time) = 500 ms/20 ms, echo train length 4, acquisition bandwidth 20 kHz, no signal averaging, cylindrical k-space coverage. The second experiment was carried out with a different set of parameters: FoV $180\times 240 \times 180$ $\hbox {mm}^3$, $1.5 \times 1.5 \times 3$ $\hbox {mm}^3$, $T_R$/$T_E$ = 400 ms/20 ms, echo train length 5, acquisition bandwidth 20 kHz, no signal averaging. All methods were carried out in accordance with relevant guidelines and regulations.

## Results

**Figure 4:**
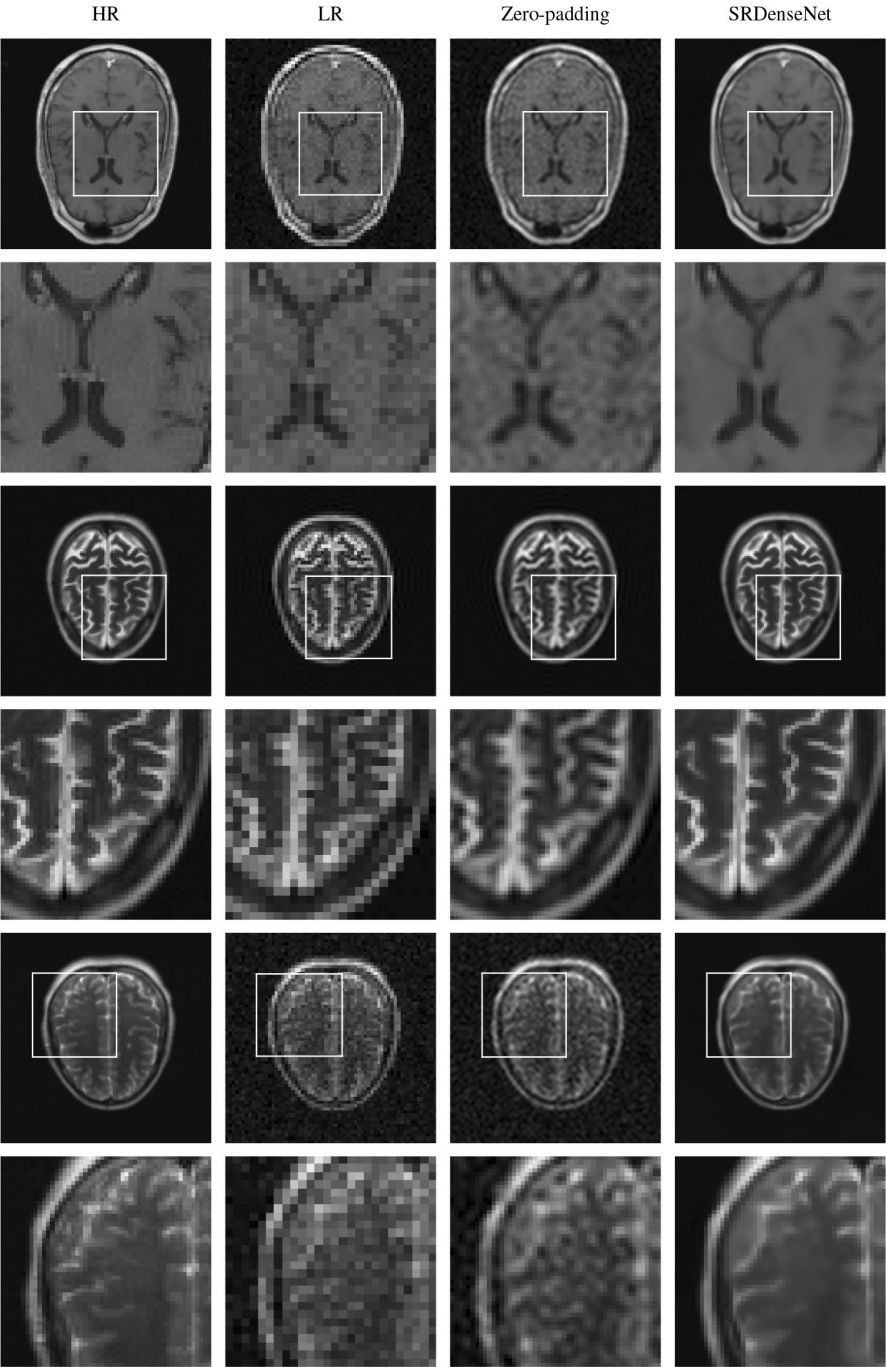
In the first column, we have three different HR images (which are high-field MR images from the database) from our test set. These HR images are our reference images, i.e., in an ideal scenario, the network would generate these exact images. The first, third and fifth rows show the full images, in the second, fourth and sixth rows we find zoomed-in versions of patches of the images in the first, third and fifth rows. The second column shows the noisy LR images corresponding to the HR images in the first column. These images are fed into the trained convolutional neural network. The third column shows the SR images obtained by zero-padding k-space, and in the fourth column, we see SR images obtained by applying SRDenseNet.

To get some idea of what image quality we can expect when we apply SRDenseNet to noisy LR images, we first focus on the output of the network when we present it with high-field MR images that were artificially down-sampled and contaminated with noise. To this end, we use images from the test set. In the first column of Fig. 4, HR images (of $128\times 128$ pixels) of three different brains are shown. The corresponding LR images (of $64\times 64$ pixels), which we obtained by eliminating the high-frequency components in k-space using Eq. (1), are shown in the second column. By zero-padding the k-space data corresponding to the LR image to a size of $128\times 128$, the images in the third column were obtained. Additionally, the LR images were fed into the trained convolutional neural network, resulting in the images in the fourth column. We observe that using a convolutional neural network can improve image quality. The images produced by the network are sharp and most of the structures that are present in the HR images are recovered. The peak signal-to-noise ratio (PSNR) and structural similarity index (SSIM) values of the images obtained using zero-padding and using SRDenseNet are shown in Table 1. As expected, both metrics increase when the network is applied, which indicates an improvement in image quality.

**Table 1:** PSNR and SSIM values of the reconstructions obtained by zero-padding the k-space data for and by applying SRDenseNet to the three different MR brain images shown in Fig. 4.

	Zero-padding (PSNR/SSIM)	SRDenseNet (PSNR/SSIM)
First brain	35.39/0.9506	41.79/0.9875
Second brain	37.14/0.9706	45.47/0.9953
Third brain	36.54/0.9265	42.69/0.9876

**Figure 5:**
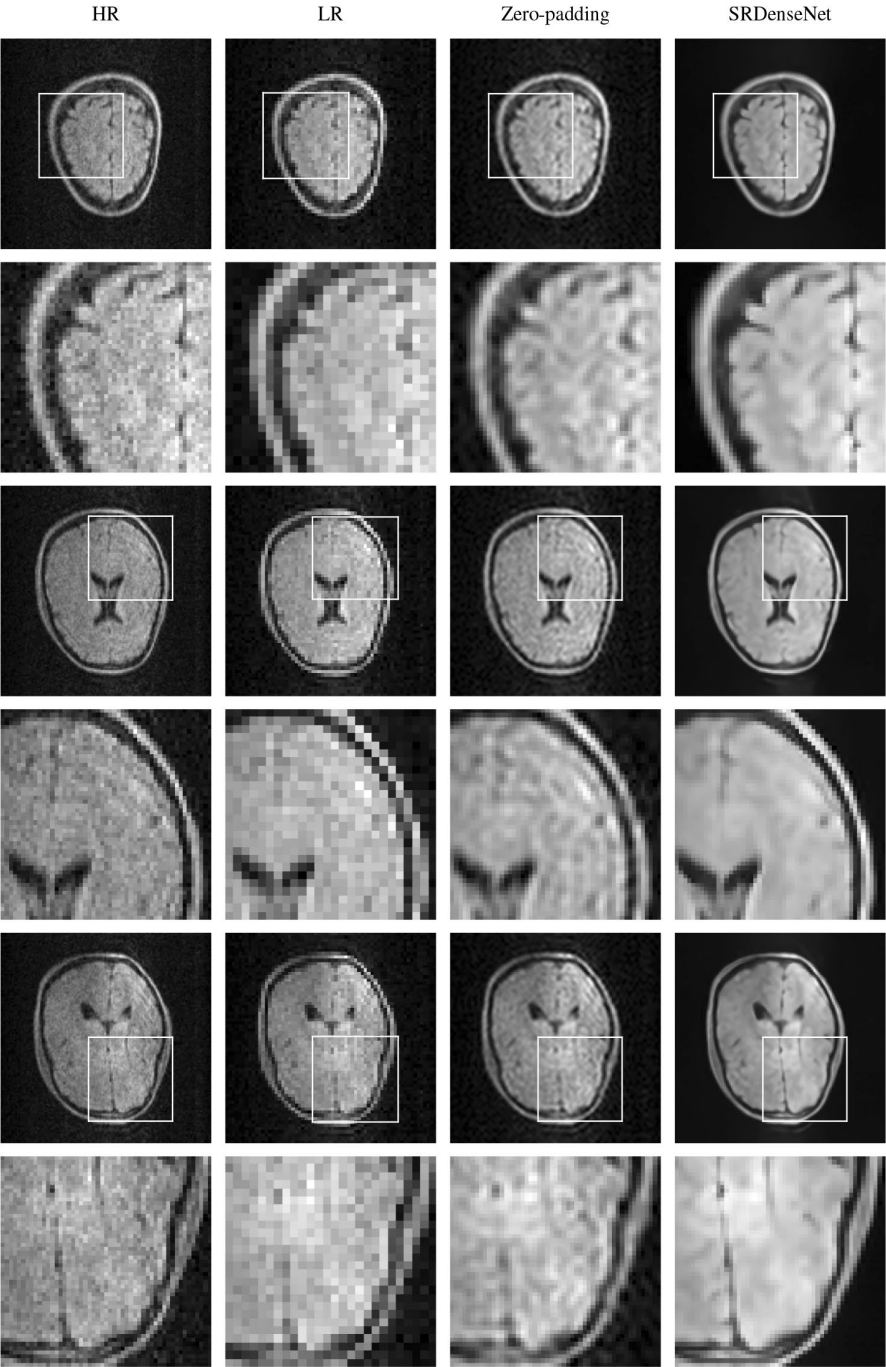
In the first column, we have three different HR slices of a brain image acquired using a low-field MRI scanner. These HR images are our reference images. The first, third and fifth rows show the full images, in the second, fourth and sixth rows we find zoomed-in versions of patches of the images in the first, third and fifth rows. The second column shows the LR images corresponding to the HR images in the first column. These images are fed into the trained convolutional neural network. The third column shows the SR images obtained by zero-padding k-space, and in the fourth column, we see SR images obtained by applying SRDenseNet.

Three slices, of $128\times 128$ pixels, of the first low-field image acquired are shown in the first column of Fig. 5. (We note that, since cylindrical k-space coverage was employed, the HR image could have been of a slightly better quality. However, we believe it suffices for our purposes.) The second column shows the corresponding $64\times 64$ pixel LR images, which were obtained after eliminating the high-frequency components from k-space, using Eq. (1), and adding noise. These images are used as the network input. The third and fourth column show the images obtained after zero-padding the k-space data (corresponding to the LR image) and the results obtained using SRDenseNet. We observe that for these low-field LR images, a convolutional neural network can help improve image quality. The edges are sharp and high-frequency components seem to be recovered to a large extent.

**Figure 6:**
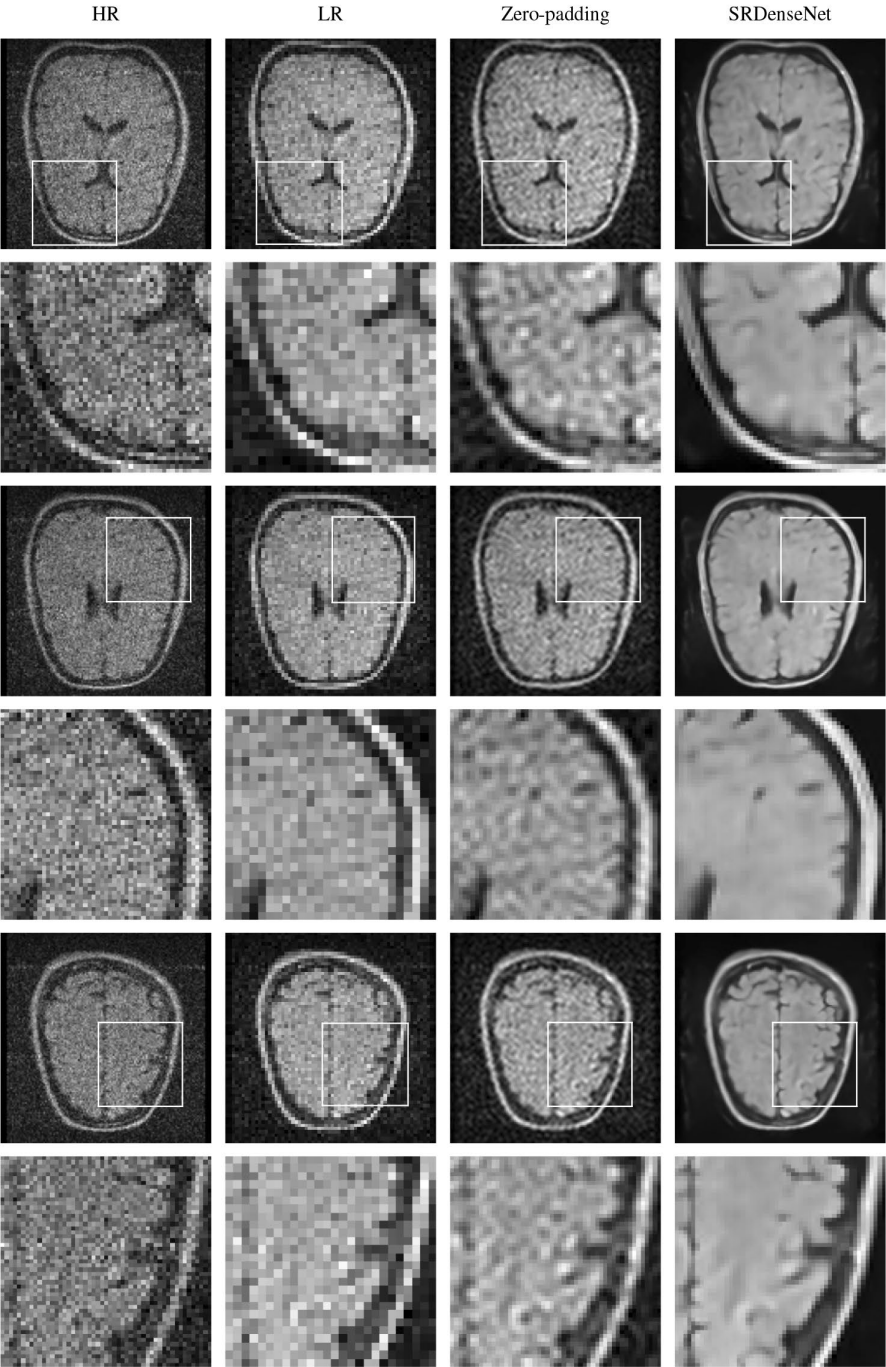
In the first column, we have three different HR slices of a second brain image acquired using a low-field MRI scanner. Again, these HR images are our reference images. The first, third and fifth rows show the full images, in the second, fourth and sixth rows we find zoomed-in versions of patches of the images in the first, third and fifth rows. The second column shows the LR images corresponding to the HR images in the first column. These images are fed into the trained convolutional neural network. The third column shows the SR images obtained by zero-padding k-space, and in the fourth column, we see SR images obtained by applying SRDenseNet.

Figure 6 shows the $128\times 128$ pixel HR images reconstructed using the data acquired during the second experiment (note that these HR images were obtained from the original 160x120 slices by eliminating the outermost pixels in one direction and zero-padding the image in the other), the corresponding $64\times 64$ pixel LR images which are used as the input to the network, the image obtained after carrying out zero-padding in k-space and the super-resolution images generated by the network. Here, too, we see that the network yields images that are sharp and contain significantly more details than the input LR images.

## Discussion and conclusion

Scan times are long in MRI, and it is desirable to decrease scan duration as much as possible, while maintaining sufficient image quality. Super-resolution image reconstruction aims to increase the resolution of one or several LR images. In this work, we used a convolutional neural network of the SRDenseNet architecture to carry out single-image super-resolution for brain images acquired using a low-field MRI scanner. We pre-processed images from a publicly available dataset to obtain pairs of noisy LR images (which were meant to emulate low-field MR images as much as possible) and noise-free HR images, on which we subsequently trained the network. The network was shown to yield sharp super-resolution images upon being presented with noisy low-resolution images acquired using a low-field MRI scanner. We note that, by training on noisy LR images and their noise-free HR counterparts, the network has strong denoising capabilities as well. This is especially relevant for low-field MRI, compared to high-field MRI, as one of the key differences between the two is the much lower SNR we have to deal with in the former. Additionally, this approach is fast: it can be carried out in only a few seconds.

The low-field HR images that are available are somewhat contaminated by noise, which makes them different from objective ground truth images. Therefore, gauging the quality of our SR reconstructions is challenging. However, based on visual inspection, the convolutional neural network seems to capture most of the structures and details in the SR image that are present in the HR image. These results indicate that it might be possible to decrease the scan duration significantly and still reconstruct images of sufficient quality, using a deep learning-based method.

We do note that it is of the utmost importance to be cautious when it comes to the output of neural networks. While they are very powerful, it is well known that convolutional neural network are not infallible: at times they can produce artifacts or they can fail to produce significant details. We will apply this network to more low-field MR brain images, to gauge how often such errors occur. Additionally, it is vital that radiologists are included in the evaluation of the performance of our approach (or any deep learning-based medical imaging approach, for that matter).

While a convolutional neural network exists that is closely related to a 3D version of SRDenseNet^29^, we decided to first focus on super-resolution for 2D images, as the training phase requires less computation power and less memory. However, in the future, we will make extending this approach to 3D a priority. This is especially interesting for low-field MRI as 3D acquisitions have the advantage of a higher SNR.

We trained the network on MR brain images which were acquired using high-field MRI scanners. While using low-field data might have been preferable to prevent potential modeling errors, we followed this approach because a large amount of data is required to train the network and acquiring enough low-field MR images to train the network from scratch is not feasible. By adding noise to the (reduced) k-space data corresponding to each of the images in the database, we attempted to simulate low-field MRI data. When a sufficient number of low-field MR images does become available, we could use transfer learning to tailor the network even more towards low-field MR images. In transfer learning, knowledge about one task is transferred to a related task^40^. In deep learning, it can be used when the amount of data available is insufficient to train a network to carry out the task at hand, but a large dataset or a pre-trained network is available for a similar task, see for example Dar et al.^41^ In our case, we could acquire a small dataset of low-field MR images (i.e., a few dozen) and apply transfer learning to our trained network, using this small dataset.

The potential of deep learning-based techniques to address imaging bottlenecks in the field of low-field MRI was demonstrated by Koonjoo et al.^42^ They use an end-to-end deep convolutional neural network approach to boost SNR in low-field MR images acquired from highly noise-corrupted data. We note that in their paper, they describe using high-field MR data and images to train their neural network as well.

In conclusion, the main contribution of this paper is that our results show that deep learning-based methods have the potential to tackle another problem in the field of low-field MR imaging, namely how to increase the resolution of noisy LR images. We believe that, using these techniques, it is possible to bring low-field MRI technology a step closer to being used in clinical practice.
